# Is ischaemic heart failure an autoimmune disease?

**DOI:** 10.1002/ehf2.14636

**Published:** 2023-12-28

**Authors:** Susanne Sattler

**Affiliations:** ^1^ National Heart and Lung Institute, Imperial College London Hammersmith Campus, Du Cane Road London W12 0NN UK; ^2^ Department of Pharmacology Otto‐Loewi Research Center, Medical University of Graz Graz Austria; ^3^ Department of Cardiology LKH‐Univ. Klinikum Graz, Medical University of Graz Graz Austria

Inflammation and autoimmunity have been implicated beyond doubt in the pathology of myocarditis and inflammatory cardiomyopathy.^1^, ^2^ Increasing evidence now also points towards the existence of a self‐perpetuating cycle of autoimmunity and inflammation (*Figure* *1*) in post‐ischaemic heart failure. This vicious cycle starts with tissue damage in the myocardium causing acute inflammation, which may—in genetically or environmentally susceptible individuals—develop into an autoimmune response, triggering more, now autoimmune‐mediated, damage. Chronic myocardial inflammation as manifestation of established anti‐heart autoimmunity then contributes to the progression of heart failure through broad immune‐dysregulation, local inflammation and fibrosis, remodelling, and structural changes.^3^

**Figure 1:**
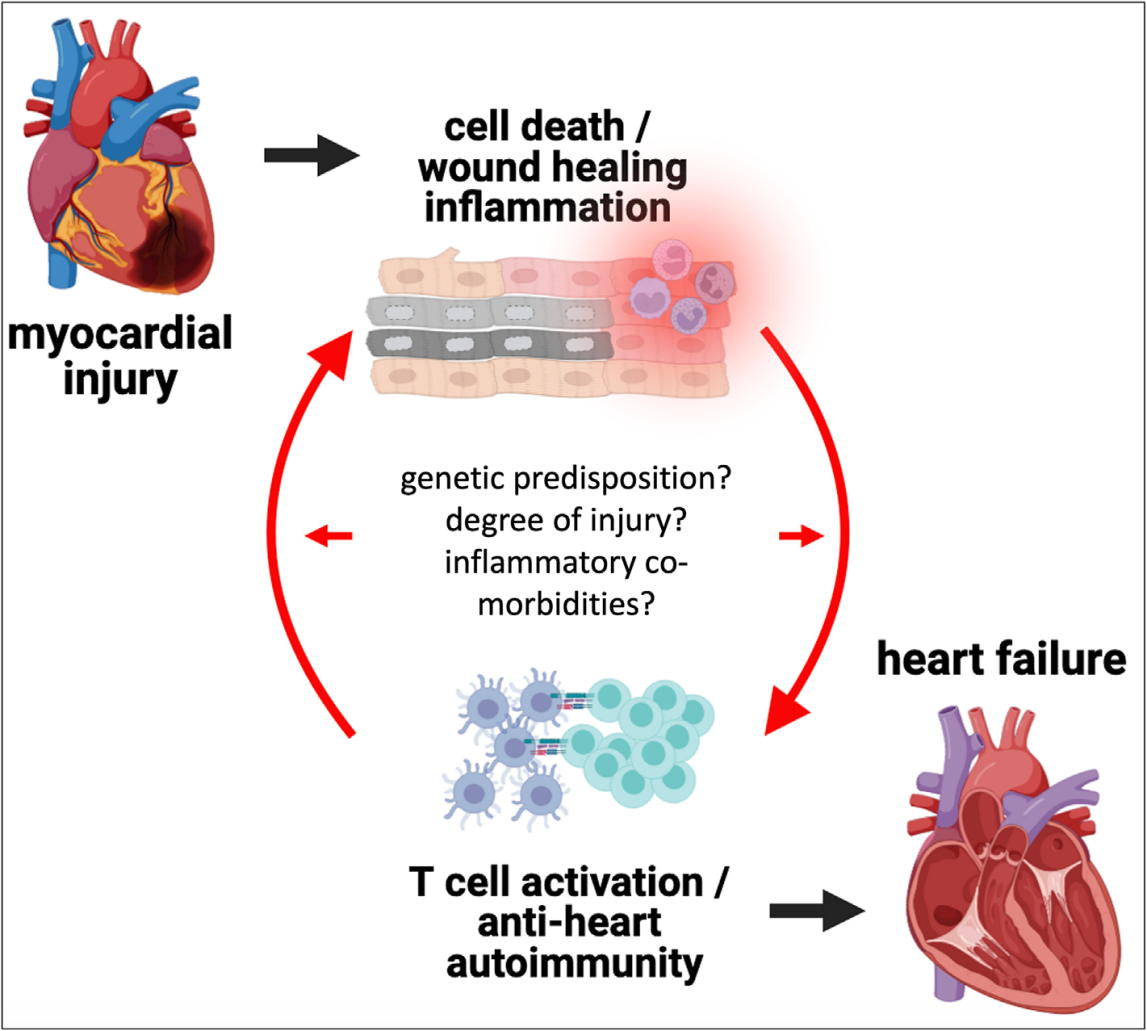
**The autoimmune‐inflammatory loop in heart failure:** Myocardial injury causes cardiomyocyte damage and necrotic cell death followed by immediate wound healing inflammation. The release of cardiac antigens in an inflammatory environment leads to an adaptive immune response, persistent anti‐heart autoimmunity, and more local inflammation. In turn, this causes more cardiomyocyte damage and drives an autoimmune‐inflammatory loop towards heart failure. Type and degree of initial injury, genetic predisposition to autoimmunity, and inflammatory co‐morbidities such as metabolic disease are likely to provide further fuel to this vicious cycle. *Source*: Image created with BioRender.com.

Massive necrotic cell death after an acute myocardial infarction (AMI) provides an obvious inflammatory insult in heart failure with reduced ejection fraction (HFrEF). While such a definite trigger is difficult to identify in heart failure with preserved ejection fraction, cardiomyocyte cell death still occurs, as indicated by raised high‐sensitivity troponin T levels.^4^ Necrosis releases cellular constituents, some of them inflammatory in themselves (‘alarmins’), and others carrying antigenic peptides capable of inducing antigen‐specific autoimmune responses. While in hindsight a lot of signs have pointed towards an autoimmune component of post‐ischaemic HFrEF for a long time, this has gathered attention only recently and begs the question ‘Should we be looking at post‐ischaemic HFrEF through the eyes of an immunologist?’

To attempt an answer, we first need to agree on the definition of autoimmunity and autoimmune disease. Tolerance against self‐antigens is normally ensured through tightly controlled selection processes during T cell development (‘central tolerance’) and ‘peripheral tolerance’ mechanisms for T cell clones escaping central tolerance. Yet, central tolerance against, for example, cardiac myosin is missing.^5^ Consequently, if peripheral tolerance mechanisms fail, get interrupted on purpose (e.g. through immune‐checkpoint inhibitor cancer therapy), or are overwhelmed by excessive inflammation (e.g. after severe tissue damage due to MI), cardiac myosin‐specific T cells can be activated to attack cardiomyocytes. Due to ‘epitope spreading’, auto‐reactive T cells will subsequently develop against a wide range of other cardiac antigens.

To label a disease as autoimmune, a set of criteria (*Rose–Witebsky's postulates*) have been suggested^6^: (1) auto‐antibodies must be detectable in all cases of the disease; (2) the disease must be experimentally reproducible by immunization with the antigen; (3) experimental disease must present with immunopathological lesions comparable to the original disease in humans; and (4) the disease phenotype must be transferable by serum or lymphoid cells.

Considering these, ischaemic tissue injury due to a blocked cardiac blood vessel is clearly not autoimmune, and autoimmunity is thus obviously not the first clinically notable cardiac event in most HFrEF patients. It is therefore critical for this discussion that we focus on heart failure, the chronic situation after the initial AMI. Once post‐AMI wound healing inflammation has subsided, any remote tissue not affected by the infarction is considered healthy. This way, it is possible to discriminate between damage obtained through the initial AMI and immune‐mediated damage due to an ongoing autoimmune‐inflammatory loop in both patients and experimental animals. Bearing in mind tis distinction between acute ischaemic vs. chronic immune‐mediated tissue damage, let us take a closer look at each of the above postulates.

1. Auto‐antibodies must be detectable in all cases of *the disease*.

A wealth of data exists on the presence and potential role of anti‐heart auto‐antibodies in various forms of heart failure, unsurprisingly in particular in heart failure of infectious or inflammatory origin.^1^ However, auto‐antibodies are also found in patients with ischaemic HFrEF, as well as rodent models of experimental MI.^7^, ^8^ The most prominent, and receiving renewed attention recently, are anti‐myosin antibodies.^9^ Due to the lack of central tolerance to cardiac myosin,^5^ its release from dying cardiomyocytes in large quantities can activate T cells, which induce B cell maturation and antibody production. However, several other cardiomyocyte auto‐antibody targets have been identified with a range of effects, including cardiac troponin,^10^ or β_1_‐adrenergic receptors.^11^ Activating auto‐antibodies against β‐adrenergic receptors exist in approximately 10% of patients with ischaemic cardiomyopathy and are associated with reduced cardiac function.^12^ Ongoing clinical trials aim at B cell depletion to prevent left ventricular dysfunction and cardiac remodelling after AMI.^13^ Importantly, while many studies understandably assess auto‐antibodies in the circulation, their pathological roles likely play out in the myocardium,^8^ where they may be able to cause functional effects or trigger complement‐ and/or cell‐mediated cytotoxicity, a classical phenomenon of established autoimmune‐mediated tissue injury. The presence of auto‐reactive T cells^14^ and the enlargement of heart‐draining lymph nodes after AMI and during heart failure^8^ are additional indirect indicators of auto‐antibody production.

The caveat to accepting this postulate is clearly that it says, auto‐antibodies must be present in *all* cases of the disease. Even if looking exclusively at post‐ischaemic HFrEF as ‘the disease’, there is considerable diversity among patients, and as auto‐antibody screening is not yet part of routine post‐MI and HFrEF monitoring, actual clinical data are still patchy. It is however conceivable that a subset of post‐MI HFrEF patients falls into an immunopathology‐high‐risk group that develop ‘autoimmune HFrEF’, not unlike what is observed for myocarditis and subsequent autoimmune cardiomyopathy.

- The disease must be experimentally reproducible by immunization with the antigen.

Here again, we need to return to cardiac myosin. Immunization with cardiac myosin provokes an anti‐heart immune response that leads to myocardial infiltration, inflammation, and injury, and is thus used as a standard animal model of myocarditis [experimental autoimmune myocarditis (EAM)]. Cardiac functional defects develop downstream of acute myocarditis just as they do in some human patients.^15^ While the goal of inducing EAM generally is a severe clinically manifest myocarditis, prolonged milder treatment is likely to lead to initially subclinical cardiac inflammation causing functional effects over time.

Animal models of ischaemic heart failure, largely induced through AMI surgeries, develop anti‐heart auto‐antibodies and myocardial inflammation^8^ entirely without active immunization. In this case, the AMI itself provides all necessary components of immunization. Immunization in a nutshell relies on antigenic peptides in combination with an adjuvant to activate the immune response. An AMI provides both; (1) plenty of the antigenic peptide released from necrotic cardiomyocytes^16^ and (2) a highly inflammatory environment caused by tissue damage as endogenous adjuvant.^17^

While immunization with cardiac myosin is considered a model of myocarditis, this clearly also proves that at least one of the likely culprit antigens in HFrEF induces inflammatory lesions in the myocardium. Accepting this postulate is thus a simple matter of putting one (*immunization with cardiac myosin causes inflammatory cardiac damage*) and one (*an AMI is an endogenous immunization event*) together.

- Experimental disease must present with immunopathological lesions comparable to the original disease in humans.

Both human and experimental heart failure tissue exhibits common immunopathological features. These are immune‐complex deposition, chronic inflammation, fibrosis, and remodelling.^8^, ^18^, ^19^ In fact, the autoimmune phenotype of heart failure is most obvious in these myocardial lesions in both human patients and the experimental models, which remind starkly of immunopathological lesions in target tissues of bona fide autoimmune diseases, such as the kidney in systemic lupus erythematosus. Most importantly, inflammatory changes are evident in remote myocardial regions in ischaemic heart failure.^8^ As these remote regions are not directly affected by AMI, this is a clear indicator of ongoing anti‐heart immune responses, fulfilling the requirements for this postulate.

- The disease phenotype must be transferable by serum or lymphoid cells.

Autoimmune diseases can be studied by transferring immune cells or serum from diseased individuals to healthy recipients. If the transfer causes disease manifestations in the recipient, autoimmune mechanisms are at play. Myocarditis can be induced in experimental animals through adoptive transfer of serum or lymphocytes from animals with existing myocarditis.^15^ Most importantly, lymphocytes isolated from mice with ischaemic heart disease are able to induce a cardiac phenotype in healthy recipient rats and mice, including inflammatory infiltration,^20^ fibrosis, and functional decline.^21^

Clearly there are very few studies that directly tested this postulate, but these do indicate transferability of disease phenotype, which allows us to carefully consider this postulate as fulfilled. Signs of autoimmunity are thus widely detectable in HFrEF, and a pathological role of auto‐reactivity against the myocardium is feasible. However, whether autoimmunity is indeed causative, and the actual degree of its contribution to post‐ischaemic decline of cardiac function remains to be fully elucidated. The difficulty with defining an autoimmune aetiology of heart failure underscores yet again that heart failure is simply not a uniform condition,^2^ and development of post‐ischaemic autoimmunity likely also depends on several factors, including genetic predisposition to autoimmunity, the severity of the initial tissue damage, and co‐morbidities exacerbating inflammation (*Figure* *1*). These too are phenomena often debated for classical autoimmune diseases. Another clear hint towards a critical overlap in underlying mechanisms between autoimmune diseases and heart failure is the recent successes in using anti‐inflammatory drugs in heart disease.^2^ Therefore, while it might still be too early to globally call all ischaemic heart failure an autoimmune disease, a more inter‐disciplinary approach, including the immunologist's perspective, is certainly warranted.

## Conflict of interest

None declared.

## References

[1] Caforio ALP , Pankuweit S , Arbustini E , Basso C , Gimeno‐Blanes J , Felix SB , *et al*. Current state of knowledge on aetiology, diagnosis, management, and therapy of myocarditis: A position statement of the European Society of Cardiology Working Group on Myocardial and Pericardial Diseases. Eur Heart J 2013;34:2636‐2648. doi:10.1093/eurheartj/eht210 23824828

[2] Van Linthout S , Tschöpe C . The quest for antiinflammatory and immunomodulatory strategies in heart failure. Clin Pharmacol Ther 2019;106:1198‐1208. doi:10.1002/cpt.1637 31544235

[3] Riehle C , Bauersachs J . Key inflammatory mechanisms underlying heart failure. Herz 2019;44:96‐106. doi:10.1007/s00059-019-4785-8 30715565 PMC6439138

[4] Brouwers FP , de Boer RA , van der Harst P , Voors AA , Gansevoort RT , Bakker SJ , *et al*. Incidence and epidemiology of new onset heart failure with preserved vs. reduced ejection fraction in a community‐based cohort: 11‐year follow‐up of PREVEND. Eur Heart J 2013;34:1424‐1431. doi:10.1093/eurheartj/eht066 23470495

[5] Lv HJ , Havari E , Pinto S , Gottumukkala RVSRK , Cornivelli L , Raddassi K , *et al*. Impaired thymic tolerance to α‐myosin directs autoimmunity to the heart in mice and humans. J Clin Invest 2011;121:1561‐1573. doi:10.1172/JCI44583 21436590 PMC3069776

[6] Rose NR , Bona C . Defining criteria for autoimmune diseases (Witebsky's postulates revisited). Immunol Today 1993;14:426‐430. doi:10.1016/0167-5699(93)90244-F 8216719

[7] Youker KA , Assad‐Kottner C , Cordero‐Reyes AM , Trevino AR , Flores‐Arredondo JH , Barrios R , *et al*. High proportion of patients with end‐stage heart failure regardless of aetiology demonstrates anti‐cardiac antibody deposition in failing myocardium: Humoral activation, a potential contributor of disease progression. Eur Heart J 2014;35:1061‐1068. doi:10.1093/eurheartj/eht506 24375073

[8] Sintou A , Mansfield C , Iacob A , Chowdhury RA , Narodden S , Rothery SM , *et al*. Mediastinal lymphadenopathy, class‐switched auto‐antibodies and myocardial immune‐complexes during heart failure in rodents and humans. Front Cell Dev Biol 2020;8:695. doi:10.3389/fcell.2020.00695 32850816 PMC7426467

[9] O'Donohoe TJ , Schrale RG , Sikder S , Surve N , Rudd D , Ketheesan N . Significance of anti‐myosin antibody formation in patients with myocardial infarction: A prospective observational study. Heart Lung Circ 2019;28:583‐590. doi:10.1016/j.hlc.2018.03.008 29653839

[10] Leuschner F , Li J , Goser S , Reinhardt L , Ottl R , Bride P , *et al*. Absence of auto‐antibodies against cardiac troponin I predicts improvement of left ventricular function after acute myocardial infarction. Eur Heart J 2008;29:1949‐1955. doi:10.1093/eurheartj/ehn268 18556712

[11] Freedman NJ , Lefkowitz RJ . Anti‐β_1_‐adrenergic receptor antibodies and heart failure: Causation, not just correlation. J Clin Invest 2004;113:1379‐1382. doi:10.1172/JCI21748 15146232 PMC406535

[12] Jahns R , Boivin V , Siegmund C , Inselmann G , Lohse MJ , Boege F . Autoantibodies activating human β_1_‐adrenergic receptors are associated with reduced cardiac function in chronic heart failure. Circulation 1999;99:649‐654. doi:10.1161/01.CIR.99.5.649 9950662

[13] Mallat Z . Rituximab in patients with ST‐elevation myocardial infarction (RITA‐MI2). 2023. Available from: https://clinicaltrials.gov/study/NCT05211401. Accessed 5 November 2023.

[14] Hapke N , Heinrichs M , Ashour D , Vogel E , Hofmann U , Frantz S , *et al*. Identification of a novel cardiac epitope triggering T‐cell responses in patients with myocardial infarction. J Mol Cell Cardiol 2022;173:25‐29. doi:10.1016/j.yjmcc.2022.09.001 36122767

[15] Błyszczuk P . Myocarditis in humans and in experimental animal models. Front Cardiovasc Med 2019;6: doi:10.3389/fcvm.2019.00064 PMC6532015 31157241

[16] Isobe M , Nagai R , Ueda S , Tsuchimochi H , Nakaoka H , Takaku F , *et al*. Quantitative relationship between left ventricular function and serum cardiac myosin light chain I levels after coronary reperfusion in patients with acute myocardial infarction. Circulation 1987;76:1251‐1261. doi:10.1161/01.CIR.76.6.1251 3677350

[17] Rock KL , Kono H . The inflammatory response to cell death. Annu Rev Pathol Mech Dis 2008;3:99‐126. doi:10.1146/annurev.pathmechdis.3.121806.151456 PMC3094097 18039143

[18] Forte E , Perkins B , Sintou A , Kalkat HS , Papanikolaou A , Jenkins C , *et al*. Cross‐priming dendritic cells exacerbate immunopathology after ischemic tissue damage in the heart. Circulation 2021;143:821‐836. doi:10.1161/CIRCULATIONAHA.120.044581 33297741 PMC7899721

[19] Frantz S , Hundertmark MJ , Schulz‐Menger J , Bengel FM , Bauersachs J . Left ventricular remodelling post‐myocardial infarction: Pathophysiology, imaging, and novel therapies. Eur Heart J 2022;43:2549‐2561. doi:10.1093/eurheartj/ehac223 35511857 PMC9336586

[20] Maisel A , Cesario D , Baird S , Rehman J , Haghighi P , Carter S . Experimental autoimmune myocarditis produced by adoptive transfer of splenocytes after myocardial infarction. Circ Res 1998;82:458‐463. doi:10.1161/01.RES.82.4.458 9506706

[21] Forte E , Panahi M , Baxan N , Ng FS , Boyle JJ , Branca J , *et al*. Type 2 MI induced by a single high dose of isoproterenol in C57BL/6J mice triggers a persistent adaptive immune response against the heart. J Cell Mol Med 2021;25:229‐243. doi:10.1111/jcmm.15937 33249764 PMC7810962

